# Sexual assault and vulnerability to sexually transmitted infections among homeless Brazilian women: a cross sectional qualitative study

**DOI:** 10.1186/s12905-023-02723-3

**Published:** 2023-10-31

**Authors:** Nayara Gonçalves Barbosa, Lise Maria Carvalho Mendes, Fábio da Costa Carbogim, Angela Maria e Silva, Thaís de Oliveira Gozzo, Flávia Azevedo Gomes-Sponholz

**Affiliations:** 1 Federal University of Juiz de Fora, Campus Universitãrio, Rua José Lourenço Kelmer, s/n - São Pedro, Juiz de Fora, 36036-900 Brazil; 2 University of São Paulo College of Nursing at Ribeirão Preto, Campus Monte Alegre, Avenida dos Bandeirantes 3900, Ribeirão Preto, 14040-902 SP Brazil; 3 Federal University of Rio de Janeiro, R. Afonso Cavalcanti, 275 - Cidade Nova, Rio de Janeiro, 20211-130 RJ Brazil

**Keywords:** Women’s health, Sexual and reproductive health, Sexual vulnerability, Sexually Transmitted Diseases, People on the street

## Abstract

**Background:**

Homeless women have complex life conditions and are often exposed to violence, sexual exploitation, rape, prostitution, reproductive disorders, survival sex trading, unintended pregnancies and sexually transmitted infections (STIs). The aim was to explore the existence of sexual aggression and vulnerability to STIs among homeless Brazilian women.

**Methods:**

Fifteen interviews were conducted with homeless women who were admitted to a women’s shelter in a large city in Brazil. Data were collected through semi-structured interviews, audiorecorded and complemented with notes of the researcher in field diary, subjected to inductive thematic analysis and analyzed in light of the social relations of gender.

**Results:**

Two themes were constructed: “Being a woman on the streets: a look at gender inequalities” and “Pain and the raped body: the scars of homeless women”. The transgression of women’s rights was observed with reports of sexual abuse interspersed with physical violence. Unprotected sexual practices were part of the daily lives of these women, with repercussions for their exposure to sexually transmitted infections. Dependence on psychoactive substances was mentioned, and transactional sex was used as a source of income to maintain such dependence, as well as to promote the women’s livelihood.

**Conclusion:**

Homeless women experience complex situations on the streets involving exposure to different types of violence, the use of transactional sex as a survival strategy and unprotected sexual practices. Furthermore, the way in which women have been exposed to sexual assault and their coping mechanisms to those require attention. Interventions are need to improve the healthcare assistance of homeless women victims of sexual assault, considering the vulnerability of this population.

## Introduction

Homelessness and housing precariousness represent social and public health problems that have a significant impact on the health and well-being of this population [1]. It is estimated that 154 million people, approximately 2% of the global population, are homeless [2]. In Brazil, there has been a significant increase in the number of people with this condition, from 96,560 to 2012 to 221,869 in 2020, an increase of 230%. However, these numbers may be underestimated due to a lack of records [3]. Women living on the streets in Brazil account for 18% of this population; the majority being black or brown, with low education and they are aged between 18 and 45 years old, in addition to being young, they are in the reproductive period [4].

The social environment of the streets is complex, stigmatizing and permeated by gender-based power imbalances [5, 6]. In this context, women are subject to violence, sexual exploitation, rape, prostitution, reproductive disorders, and a greater risk of sexually transmitted infections (STI) and unintended pregnancy, contributing to the perpetuation of the cycle of poverty and misery, in addition to unsafe abortion practices [6–9]. Among their numerous deprivations on the streets, sex can be configured as a means of meeting the subsistence needs of these women in a context of marginalization [10]. The term *survival sex trading* (SST) refers to the exchange of sex for food, money, shelter, drugs or other necessities and is a frequent practice among homeless women and other populations in a situation of social vulnerability [10].

People experiencing homelessness has a higher vulnerability to exposure to sexual violence than general population [11]. The sexual aggression rates are higher among women than men and even more expressive among the transgender homeless population [11]. Pregnancy rates due to rape are substantially higher among homeless women than the general population, with a tendency to increase amid housing instability [8]. The homeless population is also vulnerable to (STIs) due to unprotected sexual practices [8] and has a high prevalence compared to the general population, reaching 52.5% [1].

A study conducted in Baltimore, United States, evaluated the reproductive health status and needs of 70 homeless women. Among the total number of participants, 70.3% wanted to avoid pregnancy, although 57.4% did not use any contraceptive method and 16.4% reported reproductive coercion by their partner [12]. Another study conducted in seven cities of the United States with 1405 homeless, showed that only 29% victims of sexual abuse received a post − sexual assault examination, which represents a critical gap in health care delivery [11].

Thus, women who experience homelessness have unique sexual and reproductive health needs [7] that represent challenges for health services and systems [9]. However, these women have less access to essential sexual and reproductive health services than the general population [7]. In Brazil, the Street Outreach Office (*Consultório na Rua*) has been implemented to articulate and provide comprehensive health care for the homeless population, developing actions *in loco*, in street settings. The service incorporates disease prevention and health promotion practices and the expansion of access to care networks [13].

Thus, it is relevant to explore the experiences of homeless women regarding the aspects related to their sexual and reproductive health. Therefore, the aim of the present study was to explore the existence of sexual aggression and vulnerability to sexually transmitted infections among homeless Brazilian women. It is assumed that the conditions in which men and women live are not products of a biological destiny but, above all, of social constructions [13]. This perspective goes beyond gender issues, which classify biological sex as a primary form of the constitution of social relations of power and domination [14], and suggests that the various oppressions and exploitations that arise, expressed in the lives of individuals, are structurally determined by the social relations of sex, race and social class [15, 16]. Thus, homeless women are exposed, in an intertwined and dialectical manner, to the multiple expressions of the social issues manifested through the dimension of inequality. The results of this study may contribute to raising awareness and to targeting strategies for the sexual and reproductive health of homeless women, considering the intersections of gender and social class in health.

## Methods

### Study design

Cross-sectional, descriptive, exploratory survey of homeless women to assess their experiences with regard to sexual and reproductive health. This study entailed a qualitative approach with analysis from the perspective of social gender relations [14].

Study participants and setting.

The study was conducted in a foster home for women in vulnerable situations in a large municipality of the state of São Paulo, Brazil, from May to December 2021. This female foster home is characterized as a place of transitory residence, and it is necessary to carry out a screening process through social assistance to take advantage of the service. In this temporary housing, cisgender or transgender women have access to shared rooms, laundries, food, socioeducational activities and to health and social assistance services, with the objective of promoting social reintegration.

The selection criteria included homeless women, over 18 years of age, cisgender or transgender, oriented in terms of time, space and person and in the absence of evidence of acute mental health events and psychotic hallucinations. To ensure that participants were asked questions regarding orientation related to time (day, month, year, time of day), space (where are you now, in which city) and person (who she is, what her name is). Shelter professionals ensured the absence of acute mental health events and psychotic hallucinations. Concerning not included women who presented acute mental health events and psychotic hallucinations, authors understood that thoughts and perceptions during the acute episode are composed of distorted beliefs about reality, erroneous symmetries, which - despite being intertwined with the psychological suffering caused by their experiences until arriving on the streets and living on the streets - could compromise the objective of the study.

Women living on the streets were considered to be those in extreme poverty, with interrupted or weakened family ties, in the absence of regular conventional housing, with the use of public places and degraded areas as living and livelihood spaces, as well as shelter units for temporary overnight stays or temporary housing [4].

### Data collection

The interviews were conducted individually in a private room at the foster home. The first author (NGB) conducted the interviews and had previous experience interviewing and in qualitative report writing and was accompanied by an undergraduate nursing student. The researcher had had previous contact with the study population through voluntary health education activities with these women.

A semistructured script composed of two parts was used: the first part referred to the sociodemographic characterization of the participants, such as their age, race, education and time living on the streets; the second part consisted of open questions related to the experiences of women living on the streets. The latter followed the guiding question “Tell me a little about what it is like to be a woman and live on the streets”. Field notes were made during the interview and immediately afterwards in a reflexive diary.

The decision to interrupt the recruitment of new participants to complete the data collection was supported by the saturation point, understood as the stage of research development in which the data begin to repeat themselves, and this dataset allows us to respond to the focal objective of the study [17].

### Data analysis

The interviews were audio-recorded in MP3 format, had an average duration of 60 min, and were transcribed in full. The data were subjected to inductive thematic analysis [17, 18], and the coding data was performed by two researchers. A third researcher was consulted in cases of divergence to create a consensus on the codes. The following six basic steps of inductive thematic analysis were followed: (1) familiarization with the data; (2) generation of initial codes; (3) search for themes; (4) review of topics; (5) definition and naming of themes; and (6) report production. No software was used. Subsequently, the generated codes were critically reflected through the concept of genre and the pertinent literature. (Table 1)

**Table 1:** Coding of the survey data

Initial codes	Intermediate codes	Thematic categories
Exposure to violence, discrimination and prejudice	Living conditions women on the streets	Being a woman on the streets: a look at gender inequalities
Use of illegal substances		
Survival sex trading, sexual exploitation		
Discrimination, marginalization	Vulnerabilities, gender inequality	
Power relations, submission		
Lack of protection and security		
Powerless		
Sexual assault, forced sex	The streets and exposure to sexual violence: transgression of sexual and reproductive rights	Pain and the raped body: The scars of homeless women
Physical violence, threats, risk of death		
Silences	Consequences of sexual violence for women	
Effects on the physical and mental health of women		
Unprotected sexual intercourse; exposure to STI; risk of vertical transmission of infections		

The concept of the “social gender relationship” is an analysis tool and represents a theoretical synthesis of the multiple dimensions of male domination. In the singular, it is a scientific representation that reflects the uniqueness of the logic of the social organization that constitutes the domination of women by men. This is an epistemological break with naturalism and a purely biological definition of the sexes. It refers to the participation of both sexes in the production and reproduction of these relations and forms a system that structures the social order in the same way as class and race relations [14].

Brazil is the first populous country in South America. There are remarkable structural problems, which leads to high social inequalities due to stigma related to racism, gender-based violence, and LGBT phobia [19]. The concept of “social gender relationship” [14] can contribute to reflect about the Brazilian historical issues related to colonization, slavery and patriarchal organization of society. These reminiscences reflect in the racism, prejudice, misery, violences, silence, sexual exploration, vulnerability to STIs among Brazilian homeless cisgender and transgender women.

### Trustworthiness

In line with current standards of qualitative description, the Consolidated Criteria for Reporting Qualitative Research (COREQ) were followed [20].

### Ethical considerations

This study was performed in line with the principles of the Declaration of Helsinki. The Brazilian Ethical Review Authority granted ethical approval in 2021 [protocol number 4,580,233]. All participants were offered verbal information about the objectives, risks and benefits of the study and all participants gave written informed consent. To ensure anonymity, the testimonies of the participants were identified with the letter P (participant), followed by individually assigned Arabic numerals. Audio recordings and all written documentation of the research data were stored on a password protected server, only accessible to the research group.

## Results

### Characterization of the participants

Table 2 presents the demographic characteristics of the studied women. Fifteen homeless women, of which 12 were cisgender and four were transgender. The mean age of the participants was 37 years (SD: 10.11), ranging from 23 to 51 years old. Most women were mixed-race/black (n = 13/15; 86.7%) with an incomplete primary education (n = 10/15; 66.7%). Their time living on the streets ranged from 45 days to 17 years, with a median of 15 months. Their mean age at first sexual intercourse was 14.2 years (SD: 2.8), ranging from 10 to 18 years. Most cisgender women (n = 9/11; 81.8%) had 1 to 5 children.

Most of transgender women (n = 3/4; 75.0%) was HIV positive. Everyone of transgender women related a life-time history of syphilis (n = 4/4; 100%), while 18.20% of cisgender women related syphilis (n = 2/11).

**Table 2:** Demographic characteristics of the participants

Participant	Gender	Age	Race	Education	Informed STIs*
P1	Cisgender	51	Brown	Incomplete high school	
P2	Cisgender	27	Black	Incomplete high school	
P3	Cisgender	59	Black	Incomplete Elementary School	Syphilis
P4	Cisgender	42	White	Incomplete Elementary School	
P5	Cisgender	46	Brown	Incomplete Elementary School	
P6	Cisgender	38	Brown	Completed Secondary Education	Syphilis
P7	Cisgender	23	Black	Completed Secondary Education	
P8	Transgender	27	Black	Incomplete Elementary School	HIV, syphilis, gonorrhea
P9	Cisgender	29	Brown	Incomplete Elementary School	
P10	Transgender	24	Brown	Incomplete Elementary School	Syphilis, genital warts
P11	Transgender	29	Brown	Incomplete Elementary School	HIV, syphilis
P12	Transgender	41	Brown	Incomplete Elementary School	HIV, syphilis
P13	Cisgender	43	White	Incomplete Elementary School	
P14	Cisgender	38	Black	Completed Secondary Education	
P15	Cisgender	38	Black	Incomplete Elementary School	

*No one screening test was performed. We didn’t had acess to participant’s medical records in the shelter

Analysis of the interview transcripts revealed two salient themes: Being a woman on the streets: a look at gender inequalities; and Pain and the raped body: the scars of homeless women.

Being a woman on the streets: a look at gender inequalities.

### Sub-theme: living conditions women on the streets

The expectations of a woman on the streets reflect deep gender and power inequities. She is unprotected and is exposed to physical, sexual, psychological and patrimonial violence. She is vulnerable, exposed to drugs, discrimination, stigmatization and social marginalization, without any recognized human value:

…*or she is robbed, or she is beaten, or she is raped, or she gets involved with even more drugs. You end up… sinking deeper into the drugs*. P4.

*It is complicated. She is at risk of being raped. Because like it or not, there are men who don´t respect. ‘’Oh, she really is from the street. I go there, I do the job I want with her and damned it*.” P1.

*Eventuallyyou get familiar with this kind of life, you will get used to the addiction, and one day, you will be raped in the street. (….) very sad, this is not life.* P13.

*Very painful. She is discriminated against, disrespected. She is discriminated against even during sex, people look for her knowing that if she doesn´t have (sex) right, he will hit her because she was paid. (…) Sometimes, some even die.* P12.

*…because she is abused, and the man knows how to defend himself better than a woman, much better. Woman is in no position to measure strength with a man. It’s very difficult to hit, kill (a man).* P5.

Sex is a means of survival for women, from transactional sex to obtaining the minimum resources so that they can feed themselves and maintain their use of drugs and alcohol, obtain money and meet their basic human needs, in addition to the occurrence of sexual exploitation:

*I prostituted just to survive, get high every day of the week and eat. P8*

*…offers Drugs in exchange for blowjobs. P12*

*I started using drugs, prostitution was not a choice. I had to buy food and clothes, right?I mean, that thing of force, of obligation, to raise me. P11*.

*Acondition you are humiliated. That’s it – the man gives you a plate (of food) and says ‘suck my dick’ (?)you arrived, there were two, three (guys) and you had to… you know? (have sex with all of them Life on drugs, that life…it’s a dirty, filthy life. P12*.

### Sub-theme: vulnerabilities, gender inequality

The female figure on the streets refers to the symbolic image of women associated with prostitution and on the margins of society, permeated by stigma, prejudice and discrimination. However, women are noticed and visible on the streets, and their female presence draws people’s attention. They are often sexually approached with the aim of being taken advantage of, exploited, via their vulnerable condition:

*They think you are a prostitute; they want to take advantage of your situation because you are there. Most men want to take advantage of your vulnerable situation (…), giving you a drink, inviting you to come home with them.* P7.

*Because being a woman is strange, when you’re walking, there’s someone who messes with you; they think you’re easy lay because you’re addicted to drugs. They think anyone is street hooker.* P2.

Pain and the raped body: The scars of homeless women.

### Sub-theme: the streets and exposure to sexual violence: transgression of sexual and reproductive rights

Homeless women are exposed to violence on a daily basis. The marks on their bodies represent stories of aggression, sexual abuse, attempts to kill them and the pain they have experienced on the streets. They are survivors, with scars on their skin and deeper ones in their minds and souls:

*I’m alive. I was raped, beaten, stabbed, run over. Everything you can imagine, I went through. Stabbed, raped, even beaten with a stick once. I almost died, I almost went blind. Everywhere you look on my body, there is a mark. Everywhere. Here, when they tried to kill me (pointing to scar).* P8.

The condition of women staying and sleeping on the streets is lonely, exposes them to a series of vulnerabilities, including to sexual violence. The cold often forces them to seek help and gather together to warm themselves; in these interactions, they are exposed to and may suffer sexual abuse:

*I was lying, lying on the floor. I had been lying under a bridge. I asked him, ‘’Do you want to lie down here?’’ he said ‘’yeah, it’s cold’’, and it was really cold. So, I went there, I lay down next to him, then he wanted to kiss me, I said no, no, no… I was wearing a dress, he pulled my panties off; I already said no, that I didn’t want to, right?* P4.

Women do not have their will respected; even if they refuse to perform sexual practices, they are raped:

*…no respect, no. He takes you by force. The man does not respect, doesn’t care if you’re okay. If you have a problem, they don’t respect it, no. They are quite animals at this point. I want sex, and that’s it, and I go and do it*. P1.

*Man has more strength than a drugged and drunk woman.* P4.

### Sub-theme: consequences of sexual violence for women

Sexual violence causes psychological trauma and has profound emotional repercussions for women. Violence occurs in consideration of the unequal condition of women’s strength and under conditions of greater vulnerability for women, especially amid the effect of the use of illegal substances and alcohol, which prevent them from reacting or defending themselves:

*Something unpleasant happened, without my permission, but I was in a hotel before going out. I told him to stop; he did not stop. I think someone woke up because I started talking, screaming, louder. Since then I have hated him. Then I never had sex with anyone again. Not with him, not with anyone. I am not well.* P4.

Sexual violence is perpetrated by people who are known or unknown to these women. It occurs in the midst of oppression, physical violence, the use of weapons and threats. After suffering sexual violence, these women are required to remain silent so as not to suffer retaliation:

*I have been sexually assaulted on the street. The guy came with a knife to my neck to force me (have sex). Then he left, if I looked back I would be beaten. Then, I kept quiet. Today, it doesn’t hurt to remember that anymore.* P12.

*Like when I was on the street, I also suffered abuse, these things, wow, a lot of times I had to have sex with guys like that, by force, by force, without wanting to.* P8.

*I still saw those men (who committed the sexual abuse) and I couldn’t do anything. What was I going to do? I slept on the street. If I said something there’s a chance they’d kill me in my sleep.*P8.

The greater the exposure to rape, the more vulnerable they are to acquiring unwanted pregnancies and contracting STI.

*Once, a guy asked me to turning a trick and he hit me, picked up a brick, hit my head with a brick, and he wasn’t a junkie, he wasn’t a homeless person. He did what he wanted, without a condom too.* P8.

The perpetuation of sexual violence during pregnancy exposes women to risks of STIs and the vertical transmission of infection. Failure to perform tests for the diagnosis and treatment of STIs has also been reported:

*I was raped, I was pregnant, and I was on the streets of Rio de Janeiro. Dangerous as hell, (…) and I did not take the test to see if I had contracted HIV.* P7.

Behavioral changes occur after suffering sexual violence, according to the report of a woman who had the need to use medications that acted at the central nervous system level and who stopped using them, neglecting her self-care, to remain alert and to not be vulnerable to suffering some type of sexual abuse after the use of their medication:

*I’ve suffered; I’ve suffered (sexual) abuse while doped on medication, which is why today I don’t take any medication (on the streets), because I had to take medication. That’s why, today, I don’t take any medication.* P15.

## Discussion

The present study has provided reports of cisgender and transgender women living on the streets who have experienced episodes of sexual violence, risky sexual practices and exposures to STI. The reports in this study show that physical, psychological, sexual and symbolic violence are routinely experienced by the participating women. A study conducted in Los Angeles, United States, found that the main forms of violence experienced for homeless women, in the last six months, were psychological violence (87%), physical violence without weapons (48%), physical violence involving weapons (18%), and sexual violence (18%) [21]. A higher rate of sexual assault and forced sex is found among the homeless population, to the detriment of race/ethnicity and sexual orientation, with higher rates among mixed race people, cisgender women and lesbian, gay, bisexual and transsexual populations [11]. Young transgender/expansive genders, living longer on the streets and with an earlier age of homelessness, are significantly associated with living with sexual assault and forced sex [11].

Transactional sexual practices were reported as a strategy for survival and obtaining resources by this group, including food, drugs, money, or shelter. Accordingly, the literature has estimated that the practice of transactional sex is a common and may reach 50% among homeless people [22, 23]. However, many of these people perform transactional sex against their own will, motivated by desperation and a lack of alternatives [23], as suggested in the speeches of the participants in this study.

Thus, many homeless women submit to sexual intercourse to ensure security, even if this costs them the traits of utility and obedience in terms of their bodies [24]. Transactional sex is therefore highly stigmatizing and has negative effects on the health of homeless people, increasing the risk of sexual aggression, suicide attempts, depression, criminal behavior, STIs acquisition, unplanned pregnancy, etc. [22].

Violence against women living on the streets is anchored, above all, in social sex relations in which patriarchy, power relations and the hierarchical constructions of masculinity and femininity are the predominant and generalized drivers [14]. Thus, we find that women living on the streets are even more exploited in this condition. The burden imposed by their stigma and marginalization by society, plus the socially constructed class and racial conditions, perpetuate the most diverse expressions of violence [25] that these women experience in their daily lives, from insults to the serious forms of violence seen in the testimonies described above. In addition, higher rates of STIs and substance use are found among homeless women with a history of intimate partner violence than women who have not suffered violence [1]. A body that is already systematically appropriated as a thing, as a commodity, is seen even more as a public commodity when exposed in a space expressed by pauperization and racialization, such as living on the streets.

The vulnerability of women on the streets contributed significantly to the risky sexual practices and STIs exposures in the study population. We found a higher exposure to STIs in transgender women than cisgender women. A Brazilian study showed that transgender women was eight and five times more likely to test positive for HIV and syphilis, respectively, than cisgender women and men [19]. The STIs can contribute to higher morbidity and mortality in the homeless population due the lack of care and access to health services [26].

Exchanging sex for money, food, drugs, shelter or other unmet needs contributes to an increased risk of exposure to STI/HIV [27]. A greater association between unprotected sexual practices and the risk of acquiring STIs has been detected among women with a history of sexual abuse in childhood and adulthood [1], in homelessness, with psychiatric disorders, or amid alcohol and drug use [1, 19, 27], including habits of sharing injectables, and incarceration history [1]. The practices of unprotected anal sex are also more likely among homelessness, as well as drug abuse/alcohol binging and exchanging sex for money/drugs [1].

The vulnerability of homeless women in relation to lower condom use highlights an historically unfavorable gender inequality, while their exposure to sexual violence hinders their autonomy to make decisions, including in the negotiation of protected sex. These facts do not occur in isolation and are based on the patriarchal system, which gives sexual rights to men over women, practically without restrictions; it is embodied and represents a power structure based on both ideology and physical force [16]. Women, then, are “reified”, and their bodies are appropriated by men, who have social authorization, perpetrated by a pact of social silencing to dispose of them [16].

Homeless women have limitations in their access and use of health services, including sexual and reproductive health care with regard to contraception, prenatal care, appropriate STIs treatment, and access to safe abortion [6, 8]. The nonuse of these services may be related to a lack of knowledge [8, 11], to not knowing where to go to receive assistance [11], or to a lack of health insurance [11], fear, stigma, the possibility of suffering discrimination by health professionals [8] or the fear of becoming involved in the legal system [11].

Thus, when considering the significant barriers to access to sexual and reproductive health, it is essential to develop strategies for welcoming, creating bonds with and promoting the accessibility of the health system among homeless women. The vulnerability of homeless women to STIs highlight the importance of offering HIV/STIs testing, counseling, and HIV risk prevention interventions and suggest that interventions should be tailored to the needs of specific marginalized subgroups of homeless women [28], such as sexual minorities and substance misusers [19].

Addressing STIs prevention needs of homeless women can be enhanced through the integrating sexual health, and other health references services to homeless population [1]. A Brazilian study has found that gynecological care and sexual and reproductive health care in a Street Outreach Office represent an opportunity for welcoming and comprehensive care to the women being assisted, allowing the identification and care of the health needs and different demands of this population [9].

The development of intervention strategies and health education in shelters, foster homes, and drop-in centers [11] can contribute to the recognition and acceptance of the health needs of homeless women through the integration of intersectoral and networked actions. Peer navigators and small group discussions may encourage women to seek health services in the face of the lack of trust and stigma they have experienced and promote a protective network of social support [11]. However, it is also necessary to formulate public programs and policies that promote the possibility of social restructuring in the lives of these women.

The care for homeless women extends from the historicity of welcoming the human being, of the denial of rights throughout or of a good part of life. It is essential to respect people’s subjectivity, uniqueness, suffering, frustrations and desires, especially of women who experience the cascade of power and domination relations that are socially constructed in various aspects. It is essential to understand that the situations that lead women to enter the streets are segments of a social construct that objectively demonstrate the incompetence of the state in managing the problems of social reproduction and the omission and consent of society in the face of physical, economic, psychological, and social violence, namely, the social and symbolic situation in which homeless women live [24].

The present study has limitations because it did not interview the focal women in the context of the streets but in a social shelter. However, the fact that these women were in a shelter provided a safe environment for the researcher and the study participants. We did not had access to medical records to obtain data about the health conditions and STIs. The rates of STIs may be underestimated because of shame and stigma to share this information with the researchers. Despite these limitations, the study has identified deep gender inequities, exposures to violence, abuses, the use of sex as a survival strategy, and conditions that violate the human rights of these women, demonstrating the importance of actions and public policies aimed at this population.

## Conclusion

Homeless women suffer a cascade of socially constructed disadvantages that violently affect their human, sexual and reproductive rights day in and day out. Physical, sexual, psychological and symbolic abuse were reported by the interviewees. These women were accused of selling their bodies for subsistence. Unprotected sexual practices and rape were portrayed in the daily lives of these women in the context of the streets. Furthermore, the way in which women have been exposed to sexual assault and their coping mechanisms to those assaults require attention.

It is imperative that the state, as well as Brazilian society, take appropriate measures to ensure the restructuring of the lives of these women. It is not possible to talk about sexual and reproductive health care without thinking about the provision of decent housing, quality food, training for insertion in the labor market, and psychosocial support to allow these women to deal with their traumas on the streets and foster health literacy.

## Data Availability

The datasets during and/or analysed during the current study available from the corresponding author on reasonable request.

## Abbreviations

COREEQ: Consolidated Criteria for Reporting Qualitative Research
HIV: Human Immunodeficiency Virus
STIs: Sexually Transmitted Infections
LGBT: lesbian, gay, bisexual, transgender
